# Nomogram prediction of severe risk in patients with COVID-19 pneumonia

**DOI:** 10.1017/S0950268821002545

**Published:** 2021-12-09

**Authors:** Wei Tang, Run Yao, Fang Zheng, Yaxiong Huang, Guoqiang Zhou, Ruochan Chen, Ning Li

**Affiliations:** 1 Department of Infectious Diseases, The First Hospital of Changsha, Changsha, Hunan, China; 2 Department of Blood Transfusion, Xiangya Hospital, Clinical Transfusion Research Center, Central South University, Changsha, Hunan, China; 3 National Clinical Research Center for Geriatric Disorders, Xiangya Hospital, Central South University, Changsha, Hunan Province, China; 4 Department of Infectious Diseases, Xiangya Hospital, Central South University, Changsha, Hunan, China; 5 Key Laboratory of Viral Hepatitis, Hunan Province, Changsha, China

**Keywords:** COVID-19, nomogram, prediction, SARS-CoV

## Abstract

Coronavirus disease-2019 (COVID-19) elicits a range of different responses in patients and can manifest into mild to very severe cases in different individuals, depending on many factors. We aimed to establish a prediction model of severe risk in COVID-19 patients, to help clinicians achieve early prevention, intervention and aid them in choosing effective therapeutic strategy. We selected confirmed COVID-19 patients who were admitted to First Hospital of Changsha city between 29 January and 15 February 2020 and collected their clinical data. Multivariate logical regression was used to identify the factors associated with severe risk. These factors were incorporated into the nomogram to establish the model. The ROC curve, calibration plot and decision curve were used to assess the performance of the model. A total of 228 patients were enrolled and 33 (14.47%) patients developed severe pneumonia. Univariate and multivariate analysis showed that shortness of breath, fatigue, creatine kinase, lymphocytes and h CRP were independent factors for severe risk in COVID-19 patients. Incorporating age, chronic obstructive pulmonary disease (COPD) and these factors, the nomogram achieved good concordance indexes of 0.89 [95% confidence interval (CI) 0.832–0.949] and well-fitted calibration plot curves (Hosmer–Lemeshow test: *P* = 0.97). The model provided superior net benefit when clinical decision thresholds were between 15% and 85% predicted risk. Using the model, clinicians can intervene early, improve therapeutic effects and reduce the severity of COVID-19, thus ensuring more targeted and efficient use of medical resources.

## Introduction

First reported in Wuhan in December 2019, coronavirus disease-2019 (COVID-19), caused by the severe acute respiratory syndrome coronavirus 2 (SARS-CoV-2), has rapidly spread throughout China and all over the world [1–3]. Viral genome sequencing revealed SARS-CoV-2 to be a member of the *β*-coronavirus family, which also includes the Middle East syndrome coronavirus (MERS-CoV) and severe acute respiratory syndrome coronavirus (SARS-CoV) [4, 5]. As a result of its rapid global spread and high infectiousness, the World Health Organization (WHO) declared the COVID-19 outbreak a ‘Public Health Emergency of International Concern’ (PHEIC) on 30 January 2020.

COVID-19 has the characteristics of strong infectivity and complex clinical manifestations. Despite most COVID-19 patients presenting with mild symptoms, acute lung injury, respiratory distress syndrome, multiple organ dysfunction and even death can occur in severe cases [2–6]. Initial clinical and epidemiological data have shown that around 26–33% of patients need intensive care, and the mortality rate was 4–15% [4, 7, 8]. A large-scale case study including 72 314 patients infected with COVID-19 revised the initial estimates from China, and it reported that 14% developed into severe cases, with a fatality rate of 2.3% [9]. Considering the huge population of COVID-19 cases globally, the number of severe cases has been enormous as well. Therefore, exploring risk factors to predict severe cases is crucial for early intervention and treatment. Currently, there are few reports on the evaluation of COVID-19 related risk factors at home and abroad [6, 10–13]. Our study retrospectively analysed the clinical data of 228 patients admitted to the first hospital of Changsha City and constructed a predictive model to assess severe risks for patients with COVID-19. It aimed to offer a better understanding of the disease progression occurring after SARS-CoV-2 infection and establish a basis for optimising the current therapeutic strategies.

## Methods

### Patients

All confirmed patients with COVID-19 admitted to The First Hospital of Changsha from 29 January to 15 February 2020 were included in our study. Exclusion criteria: Patients diagnosed as severe cases at admission. The First Hospital of Changsha was designated as ‘the specific hospital for the treatment of patients with COVID-19 in Changsha’ by the government during the epidemic. All patients are required to undergo SARS-CoV-2 RNA screening when they were in a medical institution of Changsha, and once positive patients are found, they will be immediately transferred to designated hospitals for follow-up treatment. All COVID-19 patients will be stayed and followed up in the hospital until cured and the nucleic acid turn negative.

### Definitions

The diagnosis was confirmed by detecting SARS-CoV-2 RNA in nasopharyngeal swab samples using a virus nucleic acid detection kit according to the manufacturer's protocol (Shanghai Bio Germ Medical Biotechnology Co., Ltd). All confirmed COVID-19 patients were sent to COVID-19 designated hospitals (First Hospital of Changsha) for treatment. Patients were classified into non-severe (asymptomatic, mild and moderate) and severe types based on the severity of symptoms [11]. The severe type was defined according to the following criterion: (1) Respiratory distress with the respiratory rate over 30 per minute; (2) Pulse oximeter oxygen saturation ⩽93% in the resting state while breathing ambient air; (3) Arterial blood oxygen partial pressure (PaO_2_)/oxygen concentration (FiO_2_) ⩽300 mmHg (1 mmHg = 0.133 kPa).

### Data collection

We retrospectively collected the information of all patients including demographic data, clinical characteristics and laboratory parameters. The demographic data included age, gender and epidemiology (Patients exposed to Wuhan or close contact with a confirmed COVID-19 patient.). The clinical characteristics included time between onset and hospitalisation, underlying diseases (hypertension, diabetes, coronary heart disease, chronic obstructive pulmonary disease (COPD), kidney disease, cerebral infarction and liver disease) and symptom (fever, cough, shortness of breath, muscle ache, headache, dizziness, diarrhoea, fatigue, nausea and sore throat). As in the published study [14], we defined the disease onset as the earliest possible time of symptom onset, such as fever, cough, shortness of breath, muscle ache, headache, dizziness, diarrhoea, fatigue, nausea, sore throat and so on. When the earliest possible time of symptom onset could not be determined, we assumed it to be the earliest time of possible exposure. The laboratory parameters included creatine kinase isoenzyme, creatine kinase, lactate dehydrogenase, triglyceride, total cholesterol, high-density lipoprotein, low-density lipoprotein, D-dimer, leucocytes, haemoglobin, platelet count, lymphocytes, neutrophils, eosinophils, high-sensitivity C-reactive protein (h CRP), alanine aminotransferase, aspartate aminotransferase, total bilirubin, albumin, albumin /globulin, creatinine and urea nitrogen. Two medical staff independently reviewed the data to ensure the accuracy of the collected data.

### Statistical analyses

Continuous variables were described as means ± s.d. or median [interquartile range (IQR)] and categorical data were presented as numbers and percentages. The difference between the non-severe group and severe group was compared using Mann–Whitney test or *t*-test for continuous data and *χ*^2^ tests for categorical variables. When frequency in one of the groups was less than 5 Fisher's exact test was used.

Univariate and multivariate logistic regressions were performed to explore the association of clinical characteristics and laboratory parameters with the severe risk in patients with COVID-19. A backward step-down process was used to select variables in the final model for the nomogram. The receiver operating characteristic (ROC) curve was used to evaluate the discriminatory ability of the model. The calibration plot measured the relationship between the model's predicted probability and the actual probability. The Hosmer–Lemeshow (H–L) test was used to assess model calibration. Calibration of risk predictions was often visualised in calibration plots. These plots showed the observed proportion of events associated with a model's predicted risk. The observed proportions per level of predicted risk could not be directly observed. The observed event rates could be obtained after categorising the predicted risks, for example, using deciles. This was commonly done for the Hosmer–Lemeshow test. Firstly, according to the prediction model, the predicted probability of the outcome event of each individual was calculated. Secondly, the predicted probabilities were sorted from small to large, and were divided into 10 groups according to decile. Thirdly, the actual observation number and model prediction number of each group were calculated respectively. Fourthly, a graph was drawn according to the actual observations and model predictions for each group, and the *χ*^2^ value was calculated to obtain the corresponding *P* value. The *P* value >0.05 (H–L goodness-of-fit test) indicated that there was no statistical difference between the current model and the ideal perfect model, which was acceptable [15, 16]. Decision curve analysis was used to determine the clinical usefulness of the model, and the true-positive (TP) and false-positive (FP) classifications were considered at increasing decision thresholds. This methodology evaluated prediction models for their potential to improve clinical decision making. A decision curve showed the net benefit (NB) of using a model at different thresholds. The NB summed the TPs minus a weighted number of FPs: NB = (TP−*w*FP)/*n* (*n* was the total sample size; *w* was the relative weight of the harm of unnecessary testing *vs.* the benefit of identification of a carrier). The NB of the model and two reference strategies – test none or test all – was calculated. When the prediction model curve was closer to two curves (none and all), the clinical application value was smaller [17].

The statistical analyses were 2-tailed and *P* value <0.05 was considered statistically significant. All the statistical analyses were performed with R (http://www.R-project.org) and EmpowerStats software (www.empowerstats.com, X&Y solutions, Inc Boston, Boston, Massachusetts).

## Results

### Study participants

Overall, 239 consecutive confirmed patients with COVID-19 were admitted to the First Hospital of Changsha from 29 January to 15 February 2020. Of these 239 patients with COVID-19, 11 patients diagnosed as severe cases at admission were excluded. There were 228 patients enrolled in this study finally. 33 (14.47%) of these developed into severe cases by 6–15 days after admission. Among the 239 patients, 2 severe patients died before discharge and the remaining 237 patients completely recovered and were discharged (the clinical symptoms disappeared, and three nucleic acid tests were negative).

### Clinical characteristics and laboratory findings of patients

The average age in the severe group was significantly higher than in the non-severe group (54.39 ± 14.65 *vs.* 43.25 ± 16.71, *P* < 0.001). There were higher percentages of the patients in the severe group than in the non-severe group in hypertension (30.3% *vs.* 9.23%, *P* < 0.001), COPD (9.09% *vs.* 1.03%, *P* = 0.023), fever (93.94% *vs.* 61.54%, *P* < 0.001), shortness of breath (42.42% *vs.* 6.67%, *P* < 0.001), headache (18.18% *vs.* 6.15%, *P* = 0.018) and fatigue (57.58% *vs.* 27.69%, *P* < 0.001) (Table 1).

**Table 1.** Demographics and clinical features in patients with COVID-19

	Non-severe (*n* = 195)	Severe (*n* = 33)	*P*-value
Age, years	43.25 ± 16.71	54.39 ± 14.65	<0.001
Time between onset and hospitalisation, days	5 (3–8)	5 (4–7)	0.363
Gender/Male, *n* (%)	92 (47.18%)	20 (60.61%)	0.154
Epidemiology, *n* (%)	180 (92.31%)	28 (84.85%)	0.161
Underlying diseases *n* (%)			
Hypertension	18 (9.23%)	10 (30.30%)	<0.001
Diabetes	11 (5.64%)	3 (9.09%)	0.434^a^
Coronary heart disease	5 (2.56%)	2 (6.06%)	0.268^a^
COPD	2 (1.03%)	3 (9.09%)	0.023^a^
Kidney disease	1 (0.51%)	1 (3.03%)	0.269^a^
Cerebral infarction	5 (2.56%)	1 (3.03%)	1.0^a^
Liver disease	4 (2.05%)	2 (6.06%)	0.210^a^
Symptom, *n* (%)			
Fever	120 (61.54%)	31 (93.94%)	<0.001
Cough	108 (55.38%)	21 (63.64%)	0.376
Shortness of breath	13 (6.67%)	14 (42.42%)	<0.001
Muscle ache	15 (7.69%)	6 (18.18%)	0.054
Headache	12 (6.15%)	6 (18.18%)	0.018
Dizziness	8 (4.10%)	2 (6.06%)	0.641^a^
Diarrhoea	17 (8.72%)	3 (9.09%)	1.0^a^
Fatigue	54 (27.69%)	19 (57.58%)	<0.001
Nausea	7 (3.59%)	1 (3.03%)	1.0^a^
Sore throat	25 (12.82%)	2 (6.06%)	0.386^a^

COPD, Chronic obstructive pulmonary disease.

Epidemiology: patients exposed to Wuhan or close contact with confirmed COVID-19 patients.

a Calculated with Fisher's exact test.

Compared to the patients in the non-severe group, laboratory indicators significantly increased in the severe group including creatine kinase, lactate dehydrogenase, D-dimer, neutrophils percentage, h CRP, aspartate aminotransferase, creatinine and urea nitrogen. Additionally, laboratory indicators that significantly decreased in the severe group included lymphocyte percentage, eosinophils percentage, lymphocyte count, eosinophils count, albumin and albumin/globulin (Table 2).

**Table 2.** Laboratory findings in patients with COVID-19

	Normal range	Non-severe (*n* = 195)	Severe (*n* = 33)	*P*-value
Creatine kinase isoenzyme, U/l	0–16	9.80 (6.20–12.45)	11.20 (7.70–16.7)	0.064
Creatine kinase, U/l	25–170	67.4 (45.05–107.75)	86.7 (66.7–134.5)	<0.001
Lactate dehydrogenase, U/l	0–252	155.1 (133.9–197.82)	195.5 (177.9–276)	<0.001
Triglyceride, mmol/l	0.56–1.77	1.06 (0.77–1.44)	1.08 (0.76–1.21)	0.265
Total cholesterol, mmol/l	2.84–5.69	3.85 ± 0.83	3.56 ± 0.62	0.060
High density lipoprotein, mmol/l	1.14–1.91	0.85 ± 0.25	0.80 ± 0.22	0.309
Low density lipoprotein, mmol/l	1.0–3.0	2.73 ± 0.72	2.52 ± 0.54	0.129
D-Dimer, mg/l	0–0.5	0.22 (0.13–0.48)	0.36 (0.16–0.64)	0.042
Leucocytes, 10^9^/l	3.5–9.5	4.88 ± 1.85	4.32 ± 1.91	0.110
Haemoglobin, g/l	110–150	129.95 ± 16.17	130.55 ± 20.54	0.852
Platelet count, 10^9^/l	100–300	185.31 ± 63.65	167.06 ± 72.03	0.137
Lymphocytes, %	20.0–50.0	29.40 ± 10.15	21.51 ± 10.49	<0.001
Neutrophils, %	40.0–75.0	61.72 ± 10.42	71.91 ± 11.25	<0.001
Eosinophils, %	0.4–8.0	0.40 (0.10–1.10)	0.10 (0–0.4)	0.006
Lymphocytes, 10^9^/l	1.1–3.2	1.21 (0.92–1.69)	0.74 (0.62–1.05)	<0.001
Neutrophils, 10^9^/l	1.8–6.3	2.86 (2.13–3.54)	3.00 (2.01–3.68)	0.426
Eosinophils, 10^9^/l	0.02–0.52	0.02 (0–0.06)	0 (0–0.01)	0.042
h CRP, mg/l	0–8	11.69 (3.39–24.46)	38.10 (19.86–58.05)	<0.001
Alanine aminotransferase, U/l	0–40	18.69 (13.80–26.2)	22.11 (14.82–30.5)	0.261
Aspartate aminotransferase, U/l	0–45	23.27 (18.95–28.5)	30.2 (26.90–33.63)	<0.001
Total bilirubin, μmol/l	1.7–17.1	10.73 (8.19–15.37)	10.78 (7.61–16.82)	0.995
Albumin, g/l	60–80	38.84 ± 4.07	34.91 ± 4.36	<0.001
Albumin/globulin	1.2–2.5	1.56 ± 0.29	1.36 ± 0.29	<0.001
Creatinine, μmol/l	44–133	50.10 (39.54–63.54)	53.43 (41.51–61.29)	0.045
Urea nitrogen, mmol/l	1.8–7.1	4.14 (3.17–4.94)	4.45 (3.45–5.23)	0.040

h CRP, High-sensitivity C-reactive protein.

### Risk factors associated with severe in patients with COVID-19

All demographic data, clinical presentation and laboratory parameters, listed in Tables 1 and 2, are evaluated the association with the severe risk by univariate analysis. Our analysis showed variables that displayed statistical significance with *P* < 0.05 are listed in Table 3. These variables included age, hypertension, COPD, headache, shortness of breath, fever, fatigue, urea nitrogen, albumin /globulin, albumin, aspartate aminotransferase, h CRP, eosinophils, lymphocytes, D-dimer, creatinine, lactate dehydrogenase and creatine kinase were associated with the severe risk of patients with COVID-19 pneumonia (Table 3). We further processed the above 18 variables with multivariate logistic regression analysis and found these five variables including shortness of breath, fatigue, creatine kinase, lymphocytes and h CRP were independent risk factors of the severe risk of patients with COVID-19 (Table 3).

**Table 3.** Odd ratio and 95% confidence interval (CI) in univariate and multivariate analysis of severe risk factors for patients with COVID-19

Characteristic	Univariate OR (95% CI)	Multivariate OR (95% CI)
Age, years	1.04 (1.02–1.07)*	0.98 (0.94–1.03)
Hypertension		
No	Ref	
Yes	4.28 (1.76–10.38)*	
COPD		
No	Ref	Ref
Yes	9.65 (1.55–60.16)*	10.84 (0.97–120.72)
Headache		
No	Ref	
Yes	3.39 (1.17–9.78)*	
Shortness of breath		
No	Ref	Ref
Yes	10.32 (4.23–25.14)*	5.31 (1.70–16.59)*
Fever		
No	Ref	
Yes	9.69 (2.25–41.66)*	
Fatigue		
No	Ref	Ref
Yes	3.54 (1.66–7.56)*	3.06 (1.11–8.45)*
Urea nitrogen, mmol/l	1.20 (1.00–1.44)*	
Albumin /globulin	0.08 (0.02–0.34)*	
Albumin, g/l	0.79 (0.72–0.88)*	
Aspartate aminotransferase, U/l	1.06 (1.02–1.09)*	
h CRP, mg/l	1.05 (1.03–1.06)*	1.04 (1.01–1.06)*
Eosinophils, 10^9^/l	0.00 (0.00–0.001)*	
Lymphocytes, 10^9^/l	0.07 (0.02–0.23)*	0.18 (0.04–0.74)*
D-Dimer, mg/l	1.26 (0.96–1.65)	
Creatinine, μmol/l	1.01 (1.00–1.02)	
Lactate dehydrogenase, U/l	1.01 (1.01–1.02)*	
Creatine kinase, U/l	1.01 (1.00–1.01)*	1.01(1.00–1.01)*

COPD, Chronic obstructive pulmonary disease, h CRP, High-sensitivity C-reactive protein; OR, odd ratio; CI, confidence interval.

* Indicated *P* < 0.05.

### Construction and assessment of a novel predictive model

**Fig. 1.**
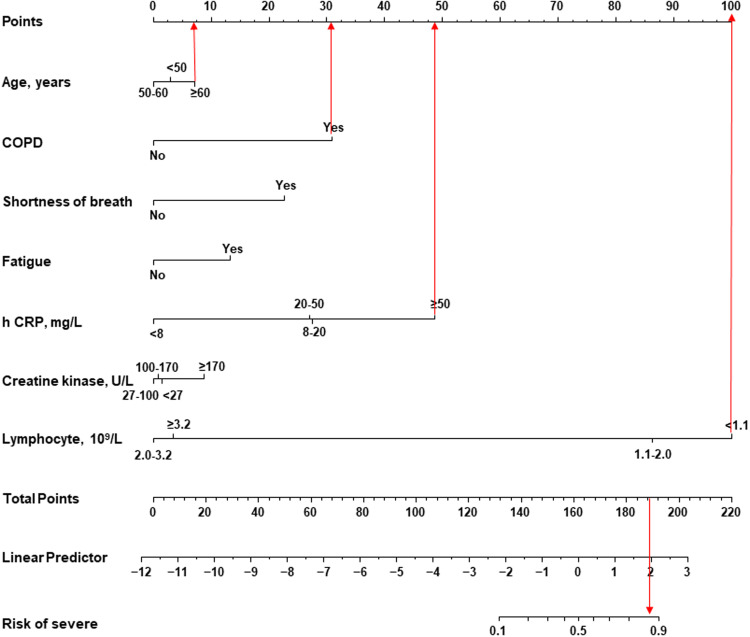
Nomogram to estimate the severe risk in patients with COVID-19.

**Fig. 2.**
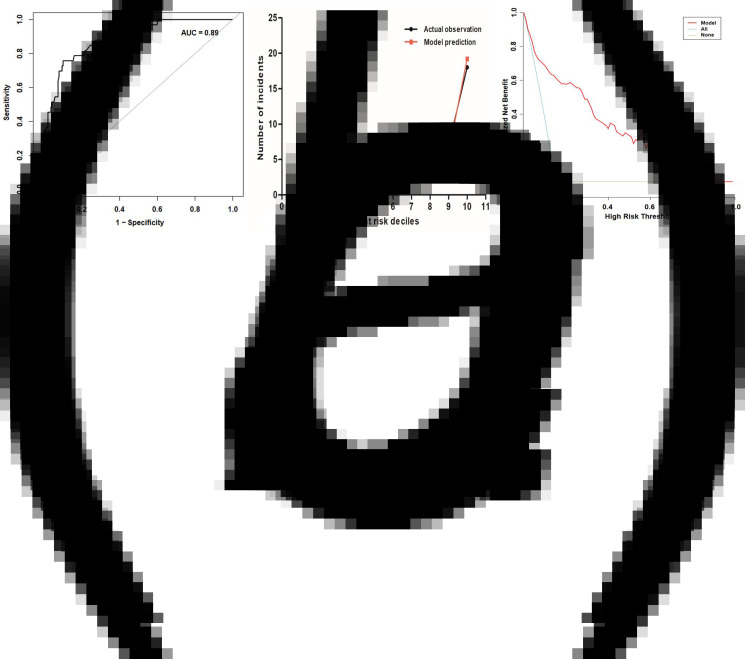
Assessment of a Novel Predictive Model (a) The ROC of the model. (b) The calibration plot of the model. (c) Decision curve analysis for the model.

The predict factors selected to formulate a predictive nomogram included age, COPD, shortness of breath, fatigue, h CRP, creatine kinase and lymphocyte (Fig. 1). Each variable corresponded to a point (top line). Assigned points for all variables were then summed to obtain total points. Once total points were located, draw a vertical line down to the bottom line to obtain the predicted probability of risk. For example, a 60-year-old (8 points) patient with COPD (32 points), his h CRP was 70 mg/l (48 points) and lymphocyte was 0.9 × 10^9^/l (100 points). This gives a total of 188 points with a corresponding risk probability of 88%. The performance of the nomogram was measured by ROC curves and the area under the curve (AUC) was 0.89 (95% CI 0.832–0.949), with a sensitivity of 75.76%, a specificity of 89.84% and an accuracy of 87.73% (Fig. 2a). The ROC curves showed that the model had good discrimination. In addition, calibration plots graphically showed the model had good calibration (Fig. 2b). The extent of agreement between the predicted probability and the actual probability of severe risk is shown in Figure 2b. The Hosmer–Lemeshow test result showed that there was no significant difference (*P* = 0.97), indicating that the predicted probability closely matched the actual probability. The decision curve demonstrated that the model had additional clinical value since it had the highest NB across a broad range of predicted probabilities ranging from 15–85% risk (Fig. 2c). This suggested that basing decisions on the model would yield an overall NB, as opposed to not using the model.

## Discussion

The rapid and extensive spread of SARS-CoV-2 infection in the world has resulted in a tremendous loss of safety in peoples’ lives [18]. Therefore, identifying risk factors on admission to predict the likelihood of disease progression, would be beneficial to physicians when they are making a reasonable decision on patient management. Our study provides comprehensive data on the epidemiological, demographic, clinical and laboratory characteristics of 228 hospitalised patients with COVID-19 in the first hospital of Changsha, which is the largest local dedicated hospital for treating COVID-19 patients. Hence, it may represent the general situation of COVID-19 infection, except for severely affected areas, such as Wuhan. In this study, shortness of breath, fatigue, creatine kinase, lymphocyte and h CRP were independent risk factors for severe risk in COVID-19 patients, while age and COPD had no significant association with the severity of COVID-19. The results of a study with 262 patients were consistent with our study [19]. This might be related to the small sample size. In Fang *et al*. 's study, they found older age and COPD associated with the severity of COVID-19 [20]. Similarly, in Xu *et al*.'s review, they observed that elderly male patients with COPD were more likely to develop severe COVID-19 infections [21]. For these reasons, five independent risk factors together with age and COPD were all selected to formulate a nomogram model to predict the severe risk of COVID-19 patients on admission. Based on these predictors, a risk nomogram with the AUC of 0.89 was established for the prediction of severe COVID-19, suggesting that our predict model had good discrimination. The good performance of this novel nomogram model was also confirmed by calibration plots and decision curves. The calibration curve demonstrated excellent consistency between the prediction of our nomogram and the observed curve. The decision curve analysis further showed that our nomogram conferred significantly high clinical NBs.

Previous studies have reported several clinical characteristics in severe cases and patients with adverse outcomes following COVID-19 infection. Older age, comorbidities such as hypertension, respiratory disease, diabetes, cancer, cardiovascular disease, high lactate dehydrogenase level and lymphocytopenia have all been associated with an increased risk of mortality [4, 9, 22–24]. Obesity and smoking have been reported to correlate with increased risks in other studies [4, 23]. In a study from Italy, men were at higher risk than women, which could be partly due to their higher smoking rates and subsequent comorbidities [25]. In our study, except for age and COPD, there were other factors included in the model, such as symptoms like shortness of breath and fatigue, laboratory data like creatine kinase, lymphocyte and h CRP. If the total points in the model added exceeded 160, the patient was noted to have a 50% risk of progressing to the severe status and perhaps requiring early intervention and more active treatment or even intensive care. The higher the points calculated, the higher the risk for the patient. The nomogram scoring system with seven clinical parameters seemed to be simpler than the 12-parameter MuLBSTA score proposed in the study by Guo *et al*. [26].

Our study also showed that the level of D-dimer was higher in the severe group than in the non-severe group. High levels of D-dimer were correlated with 28-day mortality in patients with infection or sepsis identified in the emergency department [27]. Mechanisms involved included systemic pro-inflammatory cytokine responses and local inflammation, which mediate atherosclerosis and plaque rupture, predisposing the patient to ischaemia and thrombosis. This indicates that severe patients may have a high risk of embolism, thus close monitoring and early intervention are needed [28–30]. Additionally, angiotensin-converting enzyme 2 (ACE2), the cellular receptor for SARS-CoV-2 entry, is expressed on myocytes and vascular endothelial cells [31, 32], hence there is, at least, a theoretical basis for direct cardiac and vascular involvement in SARS-CoV-2 infection. Our study showed that the severe group had elevated creatine kinase, which was possibly associated with myocardial injury, as reported in several studies [33]. Currently, the specific mechanism remains exclusive. Therefore, in patients with SARS-CoV-2 infection, cardiac and vascular damage cannot be ignored depending on the situation, and dynamic monitoring is recommended. As an acute-phase reactive protein, h CRP usually correlates positively to the severity of inflammation in many diseases. The h CRP has been used as a factor to predict the severity of patients with SARS and SARS-COV-2 previously and recently [29]. It was confirmed again in our study that h CRP level was a risk factor to predict disease severity.

There are several limitations in our study. Firstly, the nomogram model was used to predict the severe risk of COVID-19 only. Like other prediction models in literature [19–21], it can be used to identify early severe COVID-19 patients at high risk and facilitate early appropriate supportive care and medical resources use. It cannot predict other situations such as mortality risk or the duration of severity. Secondly, the sample size was relatively small. It included only 228 patients in a single centre outside Hubei province and may not be suitable for predicting the outcomes of patients in areas most severely affected by the pandemic, such as Wuhan, or regions that are experiencing large-scale outbreaks of COVID-19. Thirdly, a prospective study is required to confirm the reliability of this nomogram model. Fourthly, adding other specific markers might further improve the sensitivity and specificity of the model.

## Conclusion

We established a nomogram model of seven clinical parameters to predict the disease severity of COVID-19 on admission. Application of this model with high accuracy might be beneficial for delaying or halting the progression of the disease, which may improve therapeutic treatments, reduce the severity of COVID-19 and result in the more accurate and effective deployment of medical resources.

## Data Availability

Data analysed during the current study are available from the corresponding author on reasonable request.
